# Editorial: Inflammation and immune diseases: Molecular targets for pathogenesis and treatment

**DOI:** 10.3389/fphar.2023.1174965

**Published:** 2023-03-20

**Authors:** Fan-Rong Wu

**Affiliations:** Anhui Laboratory of Inflammatory and Immune Disease, Anhui Medical University, Hefei, China

**Keywords:** inflammation, immunity, anti-inflammatory drugs, signal pathways, target proteins

Inflammation is a hallmark of host defenses against infectious agents and injury, and also contributes to the pathophysiology of many chronic diseases. Interactions of cells of the innate and adaptive immune systems, as well as inflammatory mediators, orchestrate the process of the acute and chronic inflammation that underlie diseases of many organs. A coordinated series of common effector mechanisms of inflammation contribute to tissue injury, oxidative stress, remodeling of the extracellular matrix, angiogenesis, and fibrosis in diverse target tissues. The control of inflammatory response can help to develop novel strategies to predict disease susceptibility, target and monitor therapies, and ultimately adopt new approaches to the prevention and treatment of inflammatory and immune diseases.

There are 6 papers regarding the research on this Research Topic, including 4 original research articles and 2 reviews, reporting the pathogenesis and therapeutic targets of various inflammatory and immune diseases.

Drug-induced liver injury (DILI) is a serious clinical disease associated with reactive oxygen species (ROS) burst and subsequent inflammatory responses. However, due to the special liver structure, traditional treatments are less effective and have serious side effects. Here, Yang et al. developed Epigallocatechin-3-gallate Mo nanoparticles (EGM NPs), which are molybdenum (Mo)-based nanoparticles, after overall consideration of the pathophysiology of DILI and the advantages of nanodrugs. It is demonstrated that EGM NPs treated acetaminophen (APAP)-induced DILI by scavenging ROS and inhibiting inflammation. EGM NPs effectively scavenged various ROS and reduced cell apoptosis. More importantly, EGM NPs can treat APAP-induced DILI *in vivo*, lower the levels of indicators of liver functions in liver-injured mice, reduce the area of hepatocyte necrosis and successfully inhibit endoplasmic reticulum (ER) stress in the liver. EGM NPs also showed a certain anti-inflammatory effect by reducing infiltration of macrophages, decreasing pro-inflammatory factors and inhibiting the expression levels of inducible nitric oxide synthase (NOS2) and myeloperoxidase (MPO). Collectively, these findings suggest that EGM NPs-based nanotherapeutic is a novel strategy for the treatment of DILI.

In the article entitled “Inhibition of inflammatory liver injury by the HMGB1-A box through HMGB1/TLR-4/NF-κB signaling in an acute liver failure mouse model” by Luo et al., HMGB1-A box, a specific antagonist of HMGB1, played a protective role by inhibiting inflammatory liver injury through the regulation of HMGB1/TLR-4/NF-κB signaling in the LPS/D-GaIN-induced acute liver failure (ALF) mouse model. This study showed that HMGB1-A box inhibited the development of ALF, which may be related to preventing the extracellular release of HMGB1.

Endometriosis is a common gynecological disease, characterized by the presence of endometrial-like lesions outside the uterus. This debilitating disease causes chronic pelvic pain and infertility, for which there are few effective treatments. Chemerin is a secretory protein that acts on CMKLR1 (Chemokine-Like Receptor 1) to execute functions vital for immunity, adiposity, and metabolism. Yu et al. found that chemerin and CMKLR1 are upregulated in endometriotic lesions. Knockdown of chemerin or CMKLR1 by shRNA led to mesenchymal-epithelial transition (MET) along with compromised viability, migration, and invasion of hEM15A cells. Most importantly, 2-(α-naphthoyl) ethyltrimethylammonium iodide (α-NETA), a small molecule antagonist for CMKLR1, was shown to exhibit profound anti-endometriosis effects (anti-growth, anti-mesenchymal features, anti-angiogenesis, and anti-inflammation) *in vitro* and *in vivo*. Mechanistically, α-NETA exhibited a dual inhibition effect on PI3K/Akt and MAPK/ERK signaling pathways in hEM15A cells and murine endometriotic grafts. This study highlights that the chemerin/CMKLR1 signaling axis is critical for endometriosis progression, and targeting this axis by α-NETA may provide new options for therapeutic intervention.

He et al. showed that treatment with Forsythiaside B (FTB) increased the survival rate, ameliorated the cecal ligation and puncture (CLP)-induced inflammatory response and multiple organ dysfunction, and reduced CLP-induced pathological changes. FTB also alleviated the associated coagulopathies. Additionally, this study revealed that treatment with FTB inhibited the formation of neutrophil extracellular traps (NET) and downregulated PAD4 expression in peripheral neutrophils. The effects of FTB on coagulopathies were similar to those of monotherapy with NET or PAD4 inhibitors.

In the article entitled “EZH2: Its regulation and roles in immune disturbance of SLE” by Yang et al., EZH2, as an epigenetic regulator, has various biological functions, and its most important function is to serve as histone methyltransferase. EZH2 promotes the initiation and maintenance of immune and inflammatory responses by regulating the development, activation and differentiation of various immune cells. EZH2 is abnormally elevated in lupus and is associated with immune homeostasis imbalance and disruption of autoimmune tolerance in SLE patients. Therefore, EZH2 may serve as an important biological marker for the diagnosis and might represent a target for the treatment of SLE.

TRPV1 is a non-selective channel receptor widely expressed in skin tissues, including keratinocytes, peripheral sensory nerve fibers and immune cells. It is activated by a variety of exogenous or endogenous inflammatory mediators, triggering neuropeptide release and neurogenic inflammatory response. Xiao et al. focused on the structure and biological characteristics of the TRPV1 channel and its expression in various skin cells. Notably, TRPV1 exerts different functions in various cell types. Furthermore, a great deal of studies have demonstrated the involvement of TRPV1 in skin aging and skin inflammation through different mechanisms. Therefore, the activation or blockade of TRPV1 is a promising therapeutic strategy in the treatment of chronic inflammatory skin diseases.

The foregoing articles have studied the relationship between inflammation and immune regulation in inflammatory diseases, and explored the pathogenesis and targeted treatment of the disease. Understanding the pathological characteristics of various immune diseases can provide potential new drug targets for inflammatory immune diseases.

